# Fetal structural anomalies diagnosed during the first, second and third trimesters of pregnancy using ultrasonography: a retrospective cohort study

**DOI:** 10.1590/1516-3180.2019.026906082019

**Published:** 2020-01-13

**Authors:** Fernando Felix Dulgheroff, Alberto Borges Peixoto, Caetano Galvão Petrini, Taciana Mara Rodrigues da Cunha Caldas, Daniela Rocha Ramos, Fernanda Oliveira Magalhães, Edward Araujo

**Affiliations:** I MD. Physician, Department of Obstetrics and Gynecology, Mário Palmério Hospital Universitário (MPHU), School of Medicine, Universidade de Uberaba (UNIUBE), Uberaba (MG), Brazil.; II MD, PhD. Adjunct Professor, Department of Obstetrics and Gynecology, Mário Palmério Hospital Universitário (MPHU), School of Medicine, Universidade de Uberaba (UNIUBE), Uberaba (MG); and Adjunct Professor, Department of Obstetrics and Gynecology, Universidade Federal do Triângulo Mineiro (UFTM), Uberaba (MG), Brazil.; III MD. Physician, Department of Obstetrics and Gynecology Mário Palmério Hospital Universitário (MPHU), School of Medicine, Universidade de Uberaba (UNIUBE), Uberaba (MG), Brazil.; IV MD. Physician, Department of Obstetrics and Gynecology, Mário Palmério Hospital Universitário (MPHU), School of Medicine, Universidade de Uberaba (UNIUBE), Uberaba (MG), Brazil.; V MD. Medical Resident, Department of Obstetrics and Gynecology, Mário Palmério Hospital Universitário (MPHU), School of Medicine, Universidade de Uberaba (UNIUBE), Uberaba (MG), Brazil.; VI MD, PhD. Adjunct Professor, Department of Internal Medicine, Mário Palmério Hospital Universitário (MPHU), School of Medicine, Universidade de Uberaba (UNIUBE), Uberaba (MG), Brazil.; VII MD, PhD. Associate Professor, Department of Obstetrics, Escola Paulista de Medicina (EPM), Universidade Federal de São Paulo (UNIFESP), São Paulo (SP), Brazil.

**Keywords:** Ultrasonography, Prenatal, Prevalence, Fetal anomalies

## Abstract

**BACKGROUND:**

The prevalence of congenital abnormalities in general populations is approximately 3-5%. One of the most important applications of obstetric ultrasound is in detection of fetal structural defects.

**OBJECTIVE:**

To assess fetal structural anomalies diagnosed using ultrasound in the three trimesters of pregnancy.

**DESIGN AND SETTING:**

Retrospective cohort study at the Mário Palmério University Hospital of the University of Uberaba (Universidade de Uberaba, UNIUBE), from March 2014 to December 2016.

**METHODS:**

Ultrasound data at gestational weeks 11-13 + 6, 20-24 and 32-36 were recorded to identify fetal anomalies in each trimester and in the postnatal period. The primary outcome measurements were sensitivity, specificity, positive predictive value and negative predictive value for detection of fetal anomalies and their prevalence.

**RESULTS:**

The prevalence of anomalies detected using ultrasound was 2.95% in the prenatal period and 7.24% in the postnatal period. The fetal anomalies most frequently diagnosed using ultrasound in the three trimesters were genitourinary tract anomalies, with a prevalence of 27.8%. Cardiac anomalies were diagnosed more often in the postnatal period, accounting for 51.0% of all cases. High specificity, negative predictive value and accuracy of ultrasound were observed in all three trimesters of pregnancy.

**CONCLUSION:**

Ultrasound is safe and has utility for detecting fetal anomalies that are associated with high rates of morbidity and mortality. However, the low sensitivity of ultrasound for detecting fetal anomalies in unselected populations limits its utility for providing reassurance to examiners and to pregnant women with normal results.

## INTRODUCTION

The prevalence of congenital abnormalities in the general population is approximately 3%-5%.^1^ Since the first report of the use of ultrasound in obstetrics,^2^ this has become an important tool for detection of fetal structural defects.^3^

According to the International Society of Ultrasound in Obstetrics and Gynecology, the ideal period for screening for structural defects is the second trimester of pregnancy (weeks 18 to 22).^4^ In Brazil, the preferred period for performing ultrasound screening in the second trimester is between 20 and 24 weeks of gestation. In developed countries, the number of referrals for ultrasound examinations in the first trimester (11-13 + 6 weeks) has increased, while second-trimester ultrasound is considered to be the gold standard for detecting structural anomalies.^5^ First-trimester ultrasound has utility for confirming fetal viability and gestational age, evaluating the risk of chromosomal disorders and fetal anomalies and detecting twin pregnancy and chronicity.^4^

Evaluation of nuchal translucency in the first trimester has emerged as a tool for screening for fetal structural anomalies.^6^^,^^7^ There is an association between increased nuchal translucency and chromosomal abnormalities, particularly trisomy 21 and structural anomalies. Greater nuchal translucency has been correlated with increased risk of trisomy 21 and fetal anomalies, especially cardiac abnormalities.^6^^,^^7^

More than 80% of fetal anomalies develop before 12 weeks of gestation. Therefore, good visualization of the fetus at this stage enables early detection of structural anomalies.^8^ The ultrasound detection rates for major structural anomalies in the first and second trimesters range from 13.0% to 43.6% and from 21% to 85%, respectively.^9^^,^^10^^,^^11^^,^^12^ The overall sensitivity increases to 93% when first and second trimester ultrasound examinations are combined.^13^

Detection of structural anomalies within the gestational period makes it possible to plan interventions during pregnancy or during the immediate and early postpartum period, thereby reducing perinatal and infant morbidity and mortality.^14^^,^^15^ In addition, early detection facilitates multidisciplinary planning for maternal-fetal interventions that may be required during the gestational period and provides greater information for parents and relatives.^16^

## OBJECTIVE

The objective of the present study was to assess the fetal anomalies diagnosed using ultrasound in the first, second and third trimesters of pregnancy.

## METHODS

### Study design, setting and ethics

This retrospective cohort study evaluated prenatal ultrasound examinations performed at the Mário Palmério University Hospital of the University of Uberaba (Universidade de Uberaba, UNIUBE) from March 2014 to December 2016.

The present study was approved by the Research Ethics Committee of UNIUBE (August 24, 2017; CAAE: 73231517.9.0000.5145). The need for informed consent was waived due to the retrospective nature of the present study.

### Ultrasound evaluations and measurements

Ultrasound data were extracted using the Astraia software (Astraia Software GmbH, 2000-2015, Munich, Germany) and were divided into three groups: first trimester (11-13 + 6 weeks), second trimester (20-24 weeks) and third trimester (32-36 weeks).

The ultrasound findings were confirmed during the postnatal period through physical examination of the newborn or imaging examinations, or via necropsy in cases of death. The objective of the present study was to detect structural abnormalities using prenatal ultrasound examination; however, such examinations are unable to confirm syndromic diagnoses.

### Participants and anomaly detection

Women with singleton pregnancies in which gestational age had been established using the date of the last menstrual period and was confirmed through ultrasound in the first trimester were included in this study. Cases of major and minor anomalies identified through ultrasound, together with clinical evaluation or complementary imaging tests in the postnatal period, were included. Participants who underwent first-trimester ultrasound examinations but not second-trimester ultrasound examinations were not excluded from the present study: such participants were reevaluated during the third trimester of pregnancy. The exclusion criteria for the present study were the following: (1) cases of fetal death; (2) ultrasound examinations performed after a diagnosis of fetal anomaly was made, if no other fetal anomalies had been diagnosed during previous examinations; (3) cases of pregnant women who underwent second trimester scans only, without a third trimester scan; and (4) twin pregnancies.

Major anomalies were defined as those that were considered to be lethal, severe or moderate. Minor anomalies were defined as abnormalities that would be excluded from the European Surveillance of Congenital Anomalies registry given that their medical, functional and esthetic consequences would be minor.^17^

### Ultrasound examinations

Ultrasound examinations were performed by three experienced examiners with at least five years of experience of using a Voluson E6 device (General Electric Healthcare, Zipf, Austria) equipped with a convex volumetric transducer (RAB4-6L) and operated via the abdominal route. First, second and third-trimester scans were performed in accordance with the guidelines of the International Society of Ultrasound in Obstetrics and Gynecology.^4^^,^^18^ When required, a complementary examination via the transvaginal route was performed using a volumetric endocavitary transducer (RIC5-9-D).

In accordance with our department’s protocol, second-trimester examinations (20-24 weeks) and third-trimester examinations (32-36 weeks) were offered to all pregnant women after an initial first-trimester ultrasound examination (11-13 + 6 weeks). The follow-up for fetuses that were found to have structural defects was individualized according to the structural defect identified. The structural anomalies thus detected were divided into eight groups according to the following body systems: central nervous system; face and nape of neck; thorax; heart; gastrointestinal tract; genitourinary tract; skeleton; and others. This last group included anomalies of body systems other than those listed above.

### Sample size and statistical analysis

Case recruitment was guided by an expected 3% prevalence of fetal structural anomalies, in a screening cohort with an estimated 50% sensitivity for fetal anomaly detection. We planned to recruit approximately 600 participants in each group, to achieve a sampling error of approximately 4% for sensitivity. Thus, this sample would have 80% power to detect fetal anomalies.

The data were transferred to Excel 2010 spreadsheets (Microsoft Corp., Redmond, WA, USA) and were analyzed using the Statistical Package for the Social Sciences (SPSS) software, version 14.0 (SPSS Inc., Chicago, IL, USA). The following variables were evaluated: maternal age, weight, height, body mass index, number of pregnancies, parity, number of miscarriages, newborn weight, race, history of smoking and alcohol use, history of folic acid use before and during pregnancy, chronic diseases, diseases that started during gestation, history of consanguinity, history of structural abnormalities in previous gestations and/or in the family, type of childbirth and type and location of the structural defect.

Quantitative variables were evaluated using the Kolmogorov-Smirnov test and were presented as means and standard deviations. Categorical variables were evaluated using absolute and percentage frequencies and were presented in tables. The primary outcome measurements were the sensitivity, specificity, positive predictive value and negative predictive value for detection of fetal anomalies and their prevalence. To perform the calculations, comparison was made between the findings from the ultrasound examination and the clinical and imaging examinations on the neonate after delivery.

## RESULTS

**Figure 1.**
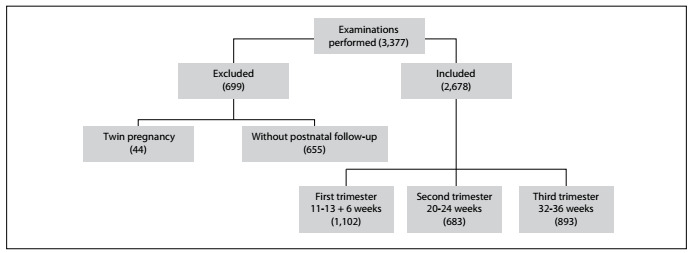
Flowchart of cases included and excluded during the study period.

A total of 3,377 ultrasound examinations were performed in the prenatal period. A total of 699 examinations were excluded: 44 due to twin pregnancies and 655 because the delivery did not take place in our service and/or the patient was lost to ­follow-up. Thus, a total of 2,678 examinations were included in the present statistical analysis. These were divided into three groups, performed at 11-13 + 6 weeks (n = 1,102), 20-24 weeks (n = 683) and 32-36 weeks (n = 893) (Figure 1).

After application of our inclusion and exclusion criteria, it was found that only 18.6% (498/2,678) of these women underwent ultrasound examination in all three trimesters. The percentages of the women who underwent ultrasound examination in the first trimester alone, second trimester alone and third trimester alone were: 17.5% (469/2,678), 8.7% (233/2,678) and 21.2% (568/2,678), respectively. However, 8.5% (228/2,678) underwent ultrasound examination in the first and second trimesters; 11.5% (308/2,678) in the first and third trimesters; and 14.0% (375/2,678) in the second and third trimesters.

The clinical and epidemiological characteristics of the study population are shown in Tables 1 and 2.

**Table 1.** Clinical characteristics of the maternal population studied

Characteristic	First trimester			Second trimester			Third trimester		
	Mean	SD	(Min-Max)	Mean	SD	(Min-Max)	Mean	SD	(Min-Max)
Age (years)	27.14	± 6.55	(14-45)	26.79	± 6.72	(14-45)	26.97	± 6.79	(13-45)
Weight (kg)	72.41	± 16.58	(40.0-141.7)	71.89	± 17.27	(40-148)	73.81	± 16.80	(40-148)
Height (m)	161.79	± 6.75	(106-180)	161.88	± 6.52	(145-180)	161.57	± 6.79	(106-180)
BMI (kg/m^2^)	27.75	± 6.32	(15.6-89.0)	27.48	± 6.32	(15.6-55.7)	28.55	± 6.98	(17.1-96.3)
Number of pregnancies	3.26	± 1.26	(2-8)	3.53	± 1.67	(2-11)	3.41	± 1.57	(2-11)
Parity	0.97	± 1.08	(0-5)	1.12	± 1.17	(0-5)	1.14	± 1.23	(0-6)
Number of abortions	1.34	± 0.75	(0.6)	1.38	± 0.96	(0-6)	1.25	± 0.79	(0-6)
Newborn weight (g)	3,035.88	± 550.7	(820-4,044)	3,081.57	± 578	(820-4,525)	3, 016.6	± 609.1	(1,410-4,525)

SD = standard deviation; Min-Max = minimum-maximum; BMI = body mass index.

**Table 2.** Ethnicity, personal antecedents, pre-existing diseases and type of delivery of the study population

Characteristics	First trimester		Second trimester		Third trimester	
	n/N	(%)	n/N	(%)	n/N	(%)
Ethnicity						
White	514/1,102	(46.6)	308/683	(45.1)	386/893	(43.2)
Black	155/1,102	(14.1)	106/683	(15.5)	137/893	(15.3)
East Asian	8/1,102	(0.7)	5/683	(0.7)	3/893	(0.3)
South Asian	0/1,102	(0)	1/683	(0.1)	1/893	(0.1)
Mixed	298/1,102	(27.0)	215/683	(31.5)	275/893	(30.8)
Not informed	127/1,102	(11.5)	48/683	(7.0)	91/893	(10.2)
Smoking						
Yes	52/1,102	(4.7)	46/683	(6.7)	83/893	(9.3)
No	997/1,102	(90.5)	630/683	(92.2)	787/893	(88.1)
Quit	1/1,102	(0.1)	1/683	(0.1)	2/893	(0.2)
Not informed	52/1,102	(4.7)	6/683	(0.9)	21/893	(2.4)
Alcohol						
Yes	10/1,102	(0.9)	13/683	(1.9)	19/893	(2.1)
No	1033/1,102	(93.7)	660/683	(96.6)	849/893	(95.1)
Quit	4/1,102	(0.4)	3/683	(0.4)	2/893	(0.2)
Not informed	55/1,102	(5.0)	7/683	(1.0)	23/893	(2.6)
Folic acid (before pregnancy)						
Yes	92/1,102	(8.3)	42/683	(6.1)	54/893	(6.0)
No or not informed	1010/1,102	(91.7)	641/683	(93.9)	839/893	(94.0)
Folic acid (during pregnancy)						
Yes	666/1,102	(60.4)	383/683	(56.1)	429/893	(48.0)
No or not informed	436/1,102	(39.6)	300/683	(43.9)	464/893	(52.0)
Diabetes mellitus						
Type 1	3/1,102	(0.3)	1/683	(0.1)	2/893	(0.2)
Type 2	4/1,102	(0.4)	3/683	(0.4)	3/893	(0.3)
GDM	59/1,102	(5.4)	38/683	(5.6)	64/893	(7.2)
Others	14/1,102	(1.3)	7/683	(1.0)	5/893	(0.6)
No or not informed	1022/1,102	(92.7)	634/683	(92.8)	819/893	(91.7)
Hypertension						
CAH	52/1,102	(4.7)	37/683	(5.4)	55/893	(6.2)
GH	7/1,102	(0.6)	8/683	(1.2)	20/893	(2.2)
Hypertensive peak	1/1,102	(0.1)	0/683	(0)	0/893	(0)
No or not informed	1042/1,102	(94.6)	638/683	(93.4)	818/893	(91.6)
Thyroid diseases						
Gestational hypothyroidism	22/1,102	(2.0)	14/683	(2.0)	15/893	(1.7)
Previous hypothyroidism	1/1,102	(0.1)	0/683	(0)	1/893	(0.1)
Hypothyroidism	96/1,102	(8.7)	66/683	(9.7)	80/893	(9.0)
Hyperthyroidism	3/1,102	(0.3)	1/683	(0.1)	1/893	(0.1)
No or not informed	980/1,102	88.9	602/683	(88.1)	796/893	(89.1)
Consanguinity						
Yes	15/1,102	(1.4)	5/683	(0.7)	8/893	(0.9)
No	874/1,102	(79.3)	575/683	(84.2)	703/893	(78.7)
Not informed	213/1,102	(19.3)	103/683	(15.1)	182/893	(20.4)
Familial or previous fetal abnormalities						
Yes	19/1,102	(1.7)	13/683	(1.9)	15/893	(1.7)
No or not informed	1083/1,102	(98.3)	670/683	(98.1)	878/893	(98.3)
Type of delivery						
Caesarean	752/1,102	(68.2)	415/683	(60.8)	557/893	(62.4)
Forceps	3/1,102	(0.3)	0/683	(0)	2/893	(0.2)
Vaginal	321/1,102	(29.1)	248/683	(36.3)	318/893	(35.6)
Not informed	26/1,102	(2.4)	20/683	(2.9)	16/893	(1.8)

GDM = gestational diabetes mellitus; CAH = chronic arterial hypertension; GH = gestational hypertension; n/N = ratio between number of participants analyzed with complete outcome and the total number of participants in each trimester of pregnancy.

The total rates of structural abnormalities detected in the prenatal period and at birth were 2.95% (79/2,678) and 7.24% (194/2,678), respectively. The rates of structural defects diagnosed using ultrasound in the first, second and third trimesters were 1.2% (13/1,102), 4.4% (30/683) and 4.0% (36/893), respectively.

**Figure 2.**
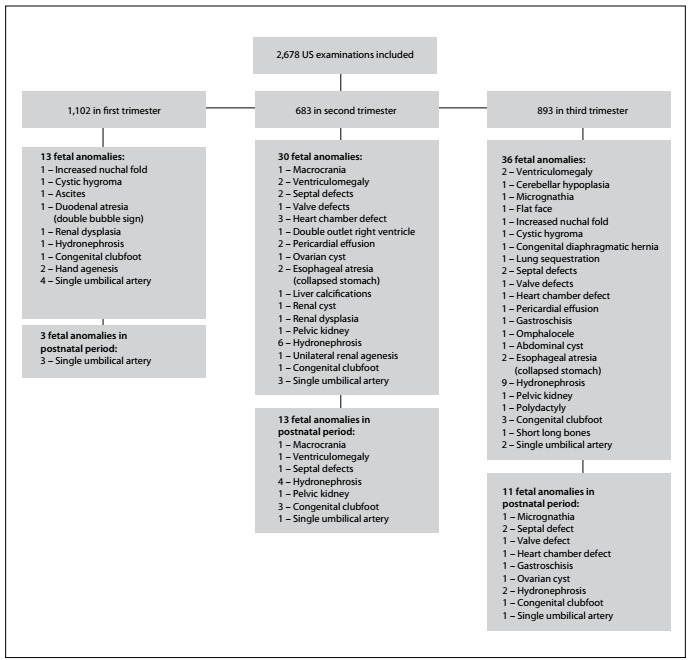
Flowchart of ultrasound (US) examinations among the cases included, according to trimester.

The most frequently diagnosed fetal anomalies in the three trimesters were in the genitourinary tract (27.9%, 22/79), heart (17.7%, 14/79), gastrointestinal tract (14.0%, 11/79), skeleton (11.4%, 9/79), single umbilical artery (11.4%, 9/79), central nervous system (7.6%, 6/79), face and neck (7.6%, 6/79) and thorax (2.5%, 2/79). In the postnatal period, cardiac abnormalities were the most common anomalies identified, accounting for 51.0% (99/194) of all cases diagnosed. The cardiac septal defects observed included atrial septal defect, ventricular septal defect and aneurysmal interatrial septum. The valve defects diagnosed included single atrioventricular valve, tricuspid insufficiency, mitral insufficiency, pulmonary insufficiency and pulmonary valve stenosis. The cardiac chamber defects included right ventricular dilatation, left ventricular enlargement, dilatation of the right chambers, right ventricular hypertrophy and hypoplastic right chambers (Table 3, Figure 2).

**Table 3.** Structural abnormalities detected using ultrasound in the three trimesters of pregnancy, according to fetal body system

Body systems/malformations	Detection rate - n (%)							Postnatal n (%)
	First trimester		Second trimester		Third trimester		Total number of US examinations	
CNS	0	(0)	3	(10.3)	3	(8.4)	6 (7.6)	17 (8.8)
Macrocrania	0	(0)	1	(3.4)	0	(0)	1 (1.3)	3 (1.6)
Ventriculomegaly	0	(0)	2	(6.9)	2	(5.6)	4 (5.0)	2 (1.0)
Hyperechoic lesions	0	(0)	0	(0)	0	(0)	0 (0)	2 (1.0)
Choroid plexus cyst	0	(0)	0	(0)	0	(0)	0 (0)	4 (2.1)
Cerebellar hypoplasia	0	(0)	0	(0)	1	2.8	1 (1.3)	0 (0)
Craniotabes/asymmetric skull	0	(0)	0	(0)	0	(0)	0 (0)	6 (3.1)
Face and nape of neck	2	(14.2)	0	(0)	4	(11.2)	6 (7.6)	17 (8.8)
Ocular hypertelorism	0	(0)	0	(0)	0	(0)	0 (0)	2 (1.0)
Dysmorphic ear	0	(0)	0	(0)	0	(0)	0 (0)	5 (2.6)
Micrognathia	0	(0)	0	(0)	1	(2.8)	1 (1.3)	2 (1.0)
Flat face	0	(0)	0	(0)	1	(2.8)	1 (1.3)	0 (0)
Increased nuchal fold	1	(7.1)	0	(0)	1	(2.8)	2 (2.5)	5 (2.6)
Cystic hygroma	1	(7.1)	0	(0)	1	(2.8)	2 (2.5)	0 (0)
Ogival palate	0	(0)	0	(0)	0	(0)	0 (0)	1 (0.5)
Cleft lip	0	(0)	0	(0)	0	(0)	0 (0)	1 (0.5)
Cleft palate	0	(0)	0	(0)	0	(0)	0 (0)	1 (0.5)
Chest	0	(0)	0	(0)	2	(5.6)	2 (2.5)	2 (1.0)
Congenital diaphragmatic hernia	0	(0)	0	(0)	1	(2.8)	1 (1.3)	0 (0)
Lung sequestration	0	(0)	0	(0)	1	(2.8)	1 (1.3)	2 (1.0)
Heart	0	0	9	(31.0)	5	(14.0)	14 (17.7)	99 (51.0)
Septal defect	0	(0)	2	(6.9)	2	(5.6)	4 (5.0)	76 (39.2)
Valve defects	0	(0)	1	(3.4)	1	(2.8)	2 (2.5)	12 (6.2)
Coarctation of the aorta	0	(0)	0	(0)	0	(0)	0 (0)	2 (1.0)
Heart chamber defect	0	(0)	3	(10.3)	1	(2.8)	4 (5.0)	7 (3.6)
Double-outlet right ventricle	0	(0)	1	(3.4)	0	(0)	1 (1.3)	1 (0.5)
Pericardial effusion	0	(0)	2	(6.9)	1	(2.8)	3 (3.8)	1 (0.5)
GIT	2	(14.2)	4	(13.7)	5	(14.0)	11 (14.0)	9 (4.7)
Gastroschisis	0	(0)	0	(0)	1	(2.8)	1 (1.3)	1 (0.5)
Omphalocele	0	(0)	0	(0)	1	(2.8)	1 (1.3)	3 (1.6)
Ascites	1	(7.1)	0	(0)	0	(0)	1 (1.3)	0 (0)
Abdominal cyst	0	(0)	0	(0)	1	(2.8)	1 (1.3)	0 (0)
Ovarian cyst	0	(0)	1	(3.4)	0	(0)	1 (1.3)	5 (2.6)
Duodenal atresia (double bubble sign)	1	(7.1)	0	(0)	0	(0)	1 (1.3)	0 (0)
Esophageal atresia (collapsed stomach)	0	(0)	2	(6.9)	2	(5.6)	4 (5.0)	0 (0)
Hepatic calcifications	0	(0)	1	(3.4)	0	(0)	1 (1.3)	0 (0)
GUT	3	(21.4)	9	(39.1)	10	(27.8)	22 (27.9)	23 (11.8)
Renal cyst(s)	0	(0)	1	(3.4)	0	(0)	1 (1.3)	3 (1.6)
Renal dysplasia	1	(7.1)	1	(3.4)	0	(0)	2 (2.5)	0 (0)
Hydronephrosis	1	(7.1)	6	(42.9)	9	(25.0)	16 (20.3)	14 (7.2)
Pelvic kidney	0	(0)	1	(3.33)	1	(2.8)	2 (2.5)	2 (1.0)
Multicystic kidneys	0	(0)	0	(0)	0	(0)	0 (0)	2 (1.0)
Enlarged kidneys	0	(0)	0	(0)	0	(0)	0 (0)	2 (1.0)
Unilateral renal agenesis	0	(0)	1	(3.4)	0	(0)	1 (1.3)	0 (0)
Skeleton	3	(21.4)	1	(3.4)	5	(14.0)	9 (11.4)	15 (7.7)
Polydactyly	0	(0)	0	(0)	1	(2.8)	1 (1.3)	2 (1.0)
Congenital clubfoot	1	(7.1)	1	(3.4)	3	(8.4)	5 (6.3)	11 (5.7)
Short long bones	0	(0)	0	(0)	1	(2.8)	1 (1.3)	0 (0)
Hand agenesis	2	(14.2)	0	(0)	0	(0)	2 (2.5)	0 (0)
Clubhand	0	(0)	0	(0)	0	(0)	0 (0)	1 (0.5)
Congenital dislocation of the knee	0	(0)	0	(0)	0	(0)	0 (0)	1 (0.5)
Others	4	(28.6)	3	(10.3)	2	(5.6)	9 (11.4)	12 (6.2)
Single umbilical artery	4	(28.6)	3	(10.3)	2	(5.6)	9 (11.4)	12 (6.2)
Total	13	(100)	30	(100)	36	(100)	79 (100)	194 (100)

US = ultrasound; CNS = central nervous system; GIT = gastrointestinal tract; GUT = genitourinary tract.

After cardiac abnormalities, the structural defects next most frequently identified in the postnatal period were in the genitourinary tract (11.8%, 23/194), central nervous system (8.8%, 17/194), face and nape of the neck (8.8, 17/194), skeleton (7.7%, 15/194), single umbilical artery (6.2%, 12/194), gastrointestinal tract (4.6%, 9/194) and thorax (1.0%, 2/194) (Table 3). The most common structural defects identified in each body system were hydronephrosis, congenital clubfoot, asymmetrical skull, left pulmonary sequestration, ovarian cyst, atrial dimorphism and single umbilical artery, respectively.

Among the 2,678 ultrasound examinations included in the present study, 79 structural abnormalities were diagnosed during the prenatal period. Twenty-five anomalies were identified through ultrasound examination and confirmed during the postnatal period, while 54 anomalies were identified through ultrasound examination but not confirmed during the postnatal period. Of the 2,678 ultrasound examinations, 2,599 were unremarkable in the prenatal period. Of these, 2,430 were unremarkable in both the prenatal and postnatal periods. In contrast, 169 anomalies were not detected through ultrasound but were identified in the postnatal period (Table 4).

**Table 4.** Number of cases diagnosed and not diagnosed through ultrasound, relative to the number of cases diagnosed in the postnatal period

	Postnatal examination (+)	Postnatal examination (-)
Ultrasound (+)	25	54
Ultrasound (-)	169	2,430

Postnatal examination (+): with fetal anomaly; Postnatal examination (-): without fetal anomaly; Ultrasound (+): with fetal anomaly; Ultrasound (-): without fetal anomaly.

The sensitivity, specificity, positive predictive value, negative predictive value, accuracy, positive likelihood ratio and negative likelihood ratio of ultrasound examinations for diagnosing structural defects in the first trimester were 14.06%, 98.65%, 39.13%, 94.90%, 93.73%, 10.0 and 0.87, respectively. The sensitivity, specificity, positive predictive value, negative predictive value, accuracy, positive likelihood ratio and negative likelihood ratio of ultrasound examinations for diagnosing structural defects in the second trimester were 27.78%, 98.14%, 45.45%, 96.06%, 94.43%, 14.6 and 0.73, respectively. The sensitivity, specificity, positive predictive value, negative predictive value, accuracy, positive likelihood ratio and negative likelihood ratio of ultrasound examinations for diagnosing structural defects in the third trimester were 23.91%, 97.76%, 36.67%, 95.94%, 93.95%, 10.9 and 0.78, respectively (Table 5).

**Table 5.** Sensitivity, specificity, positive predictive value, negative predictive value and accuracy of ultrasound in the first, second and third trimesters of pregnancy

Trimester	Sensitivity	Specificity	PPV	NPV	Accuracy	+ LH	- LH
First	14.06%	98.65%	39.13%	94.90%	93.73%	10.0	0.87
Second	27.78%	98.14%	45.45%	96.06%	94.43%	14.6	0.73
Third	23.91%	97.76%	36.67%	95.94%	93.95%	10.9	0.78

PPV = positive predictive value; NPV = negative predictive value; +LH = positive likelihood ratio; -LH = negative likelihood ratio.

## DISCUSSION

The present study evaluated the fetal structural abnormalities in an unselected population, using prenatal ultrasound in the first, second and third trimesters of pregnancy. The international classification of diseases of the World Health Organization aims to classify these structural defects according to etiology or pathogenetic mechanism.^19^ However, in the present study, structural abnormalities were classified according to the body systems in which they occurred, in order to determine the systems that were most frequently affected in an unselected population.

The prevalence of structural defects was 2.95% during the prenatal period and 7.24% during the postnatal period. Oakley et al.^1^ reported that the prevalence of fetal anomalies at birth ranged from 2% to 5% in general populations. In the United States and Europe, the rates of fetal anomalies at birth have been reported to be 3.0% and 2.4%, respectively.^20^^,^^21^ In Saudi Arabia, the prevalence of fetal anomalies at birth was reported to be 4.6%.^22^

The primary factors that affect the prevalence of fetal anomalies in different populations are consanguinity, use of assisted reproduction techniques, tobacco exposure, air pollution, water contamination and pesticide and agrochemical exposure.^23^^,^^24^^,^^25^^,^^26^^,^^27^^,^^28^ The rates of consanguinity and smoking described in Brazilian populations have been lower than those in other countries.^22^^,^^29^ We postulate that the higher prevalence of structural defects observed in the present study (higher than observed in North America, Europe and Saudi Arabia) may have arisen through assessment of cases referred to a tertiary-level healthcare center, instead of cases within a general population.

The prevalences of structural abnormality types vary according to the population assessed and the time of diagnosis. Molina-Giraldo et al.^30^ conducted a study in Bogota, Colombia, and found that the most common fetal anomalies at birth were those of the central nervous system. Sallout et al.^22^ reported that genitourinary tract anomalies were the most frequently diagnosed fetal anomalies during the prenatal period and at birth in Saudi Arabia. In the present study, genitourinary tract and cardiac anomalies were the most frequently diagnosed anomalies during the prenatal period and at birth, respectively.

The rate of detection of fetal cardiac defects has been reported to be low and dependent on the study population.^31^^,^^32^ The sensitivity of fetal echocardiography for diagnosing cardiac anomalies was found to be 33.9% in low-risk populations and 68.8% in high-risk populations,^31^ with septal defects accounting for the majority of cases of diagnostic failure.^32^ The high frequency of genitourinary tract anomalies observed in the present study may have been due to their greater ease of detection through ultrasound, in comparison with defects of other body systems such as the heart and central nervous system.

During the first trimester of pregnancy, the sensitivity of ultrasound for detecting fetal anomalies in the present study ranged from 13.0% to 43.6%. The detection rate was much higher when a detailed morphological protocol was adopted, such that up to 76.3% of major structural defects were detected. A previous study in a general population reported a first-trimester detection rate of 90% for complex congenital heart disease (either alone or in association with extracardiac abnormalities) and 69.5% for complex central nervous system anomalies.^33^ The sensitivity of ultrasound has been found to be higher in the second trimester, ranging from 21% to 85%.^9^^,^^10^^,^^11^^,^^12^

Detection of fetal anomalies in the third trimester is technically more challenging due to fetal growth, poor imaging with static ultrasound and decreased quantities of amniotic fluid.^34^ To date, few studies have evaluated the sensitivity of ultrasound for diagnosing fetal anomalies in the third trimester.^34^^,^^35^ However, this examination has an important role in identifying defects, particularly those of the central nervous system and genitourinary tract that do not develop or become evident before the third trimester.^36^^,^^37^ Manegold et al.^34^ evaluated 8,074 ultrasound examinations in a prospective study over the three trimesters of pregnancy. They found an additional 15% of fetal defects in the third trimester, especially in the genitourinary tract, heart and gastrointestinal tract. In the present study, the sensitivity of ultrasound in the first, second and third trimesters for detecting structural abnormalities was 14.06%, 27.78% and 23.91%, respectively.

According to Eureniuns et al.,^38^ variability in the reported sensitivity of ultrasound may be due to differences in the study design, type of clinical center involved, examiners’ experience and definitions used to classify the anomalies. The low sensitivity of ultrasound observed in the present study may have resulted from the inclusion of fetal anomalies such as septal and valve defects and the absence of 11 complex cardiac abnormalities, which were excluded due to a lack of postnatal results in our center’s database. These defects are often small and/or transitory.^39^ Chitty et al.^39^ indicated that only clinically significant cardiac defects should be included when assessing the ability of ultrasound to detect structural defects. Despite this recommendation, simple cardiac defects and other defects such as congenital dislocation of the knee and ogival palate were present in the present study to evaluate the limitations of ultrasound for diagnosing fetal anomalies.

The specificity, negative predictive value and accuracy of ultrasound for identifying fetal structural anomalies were high in all three trimesters of pregnancy, despite low sensitivity and positive predictive value values. These results demonstrate that ultrasound is a reliable method for confirming structural defects and for reassuring examiners and pregnant women with normal results. The low positive predictive value may be explained by the low prevalence of fetal anomalies in the present study sample.

The limitations of the present study were its exclusion of a large number of cases due to loss of follow-up and lack of postnatal results, its small number of cases with fetal anomalies and its retrospective nature. It was not possible to determine the cumulative accuracy of ultrasound, since not all the cases included had a first-trimester scan. Furthermore, first-trimester scans have not been established as routine by the Brazilian Ministry of Health.^40^ We carefully excluded from the analysis all fetal anomalies that were repeatedly reported in subsequent scans, in order to avoid inconsistencies in the accuracy of the first, second and third trimester scans.

The strength of the present study was its inclusion of an unselected population in a single center, which resulted in a high follow-up rate during pregnancy.

## CONCLUSION

In summary, ultrasound is a reliable tool for counseling the parents of children with severe fetal anomalies that are associated with high rates of morbidity and mortality. However, the low sensitivity of ultrasound in detecting fetal anomalies in unselected populations limits its utility for providing reassurance to examiners and pregnant women with normal results

## References

[1] Oakley GP (1986). Frequency of human congenital malformations. Clin Perinatol.

[2] Donald I, Brown TG (1961). Demonstration of tissue interfaces within the body by ultrasonic echo sounding. Br J Radiol.

[3] Allan L (2010). Fetal cardiac scanning today. Prenat Diagn.

[4] Salomon LJ, Alfirevic Z, Berghella V (2011). Practice guidelines for performance of the routine mid-trimester fetal ultrasound scan. Ultrasound Obstet Gynecol.

[5] Borrell A, Robinson JN, Santolaya-Forgas J (2011). Clinical value of the 11- to 13+6-week sonogram for detection of congenital malformations: a review. Am J Perinatol.

[6] Snijders RJ, Noble P, Sebire N, Souka A, Nicolaides KH (1998). UK multicenter project on assessment of risk of trisomy 21 by maternal age and fetal nuchal-translucency thickness at 10-14 weeks of gestation. Fetal Medicine Foundation First Trimester Screening Group. Lancet.

[7] Pandya PP, Goldberg H, Walton B (1995). The implementation of first-trimester scanning at 10-13 weeks’ gestation and the measurement of fetal nuchal translucency thickness in two maternity units. Ultrasound Obstet Gynecol.

[8] Jones KL, Smith DW (1997). Morphogenesis and dysmorphogenesis. Smith’s Recognizable Patterns of Human Malformation.

[9] Syngelaki A, Chelemen T, Dagklis T, Allan L, Nicolaides KH (2011). Challenges in the diagnosis of fetal non-chromosomal abnormalities at 11-13 weeks. Prenat Diagn.

[10] Hildebrand E, Selbing A, Blomberg M (2010). Comparison of first and second trimester ultrasound screening for fetal anomalies in the southeast region of Sweden. Acta Obstet Gynecol Scand.

[11] Luck CA (1992). Value of routine ultrasound scanning at 19 weeks: a four year study of 8849 deliveries. BMJ.

[12] Levi S, Schaaps JP, De Havay P, Coulon R, Defoort P (1995). End result of routine ultrasound screening for congenital anomalies: the Belgian multicenter study 1984-92. Ultrasound Obstet Gynecol.

[13] Flood K, Malone FD (2008). Screening for fetal abnormalities with ultrasound. Curr Opin Obstet Gynecol.

[14] Saari-Kemppainen A, Karjalainen O, Ylöstalo P, Heinonen O (1990). Ultrasound screening and perinatal mortality: controlled trial of systematic one-stage screening in pregnancy. Lancet.

[15] Bucher HC, Schmidt JG (1993). Does routine ultrasound scanning improve outcome in pregnancy? Meta-analysis of various outcome measures. BMJ.

[16] Crane JP, LeFevre ML, Winborn RC (1994). A randomised trial of prenatal ultrasonographic screening: impact on detection, management, and outcome of anomalous fetuses. The RADIUS Study Group. Am J Obstet Gynecol.

[17] EUROCAT Guide 1.3 and reference documents. Instructions for the Registration and Surveillance of Congenital Anomalies.

[18] Salomon LJ, Alfirevic Z, Bilardo CM (2013). ISUOG practice guidelines: performance of first-trimester fetal ultrasound scan. Ultrasound Obstet Gynecol.

[19] Organização Mundial da Saúde Classificação Estatística Internacional de Doenças e Problemas Relacionados à Saúde: CID-10.

[20] Parker SE, Mai CT, Canfield MA (2010). Updated National Birth Prevalence estimates for selected birth defects in the United States, 2004-2006. Birth Defects Res A Clin Mol Teratol.

[21] Dolk H, Loane M, Garne E (2010). The prevalence of congenital anomalies in Europe. Adv Exp Med Biol.

[22] Sallout B, Obedat N, Shakeel F (2015). Prevalence of major congenital anomalies at King Fahad Medical City in Saudi Arabia: a tertiary care centre-based study. Ann Saudi Med.

[23] Sheridan E, Wright J, Small N (2013). Risk factors for congenital anomaly in a multiethnic birth cohort: an analysis of the Born in Bradford study. Lancet.

[24] Hamdan MA, Chedid F, Bekdache GN (2015). Perinatal outcome of congenital heart disease in a population with high consanguinity. J Perinat Med.

[25] Leonardi-Bee J, Britton J, Venn A (2011). Secondhand smoke and adverse fetal outcomes in nonsmoking pregnant women: a meta-analysis. Pediatrics.

[26] Vrijheid M, Martinez D, Manzanares S (2011). Ambient air pollution and risk of congenital anomalies: a systematic review and meta-analysis. Environ Health Perspect.

[27] Hwang BF, Jaakkola JJ, Guo HR (2008). Water disinfection by products and the risk of specific birth defects: a population-based cross-sectional study in Taiwan. Environ Health.

[28] Ngo AD, Taylor R, Roberts CL (2010). Paternal exposure to Agent Orange and spina bifida: a meta- analysis. Eur J Epidemiol.

[29] Rocha RS, Bezerra SC, Lima JW, Costa FS (2013). Consumo de medicamentos, álcool e fumo na gestação e avaliação dos riscos teratogênicos [Consumption of medications, alcohol and smoking in pregnancy and assessment of teratogenic risks]. Rev Gaucha Enferm.

[30] Molina-Giraldo S, Alfonso-Ospina L, Parra-Meza C (2015). Prevalencia de malformaciones congénitas diagnosticadas por ultrasonido: tres años de experiencia en una unidad de medicina materno fetal universitaria [Prevalence in birth defects diagnosed by ultrasound: three years experience in university maternal fetal medicine unit]. Ginecol Obstet Mex.

[31] Chu C, Yan Y, Ren Y, Li X, Gui Y (2017). Prenatal diagnosis of congenital heart diseases by fetal echocardiography in second trimester: a Chinese multicenter study. Acta Obstet Gynecol Scand.

[32] Zhang YF, Zeng XL, Zhao EF, Lu HW (2015). Diagnostic Value of Fetal Echocardiography for Congenital Heart Disease: A Systematic Review and Meta-Analysis. Medicine.

[33] Iliescu D, Tudorache S, Comanescu A (2013). Improved detection rate of structural abnormalities in the first trimester using an extended examination protocol. Ultrasound Obstet Gynecol.

[34] Manegold G, Tercanli S, Struben H, Huang D, Kang A (2011). Is a routine ultrasound in the third trimester justified? Additional fetal anomalies diagnosed after two previous unremarkable ultrasound examinations. Ultraschall Med.

[35] Skråstad RB, Eik-Nes SH, Sviggum O (2013). A randomized controlled trial of third-trimester routine ultrasound in a non-selected population. Acta Obstet Gynecol Scand.

[36] Fugelseth D, Lindemann R, Sande HA, Refsum S, Nordshus T (1994). Prenatal diagnosis of urinary tract anomalies. The value of two ultrasound examinations. Acta Obstet Gynecol Scand.

[37] Malinger G, Lerman-Sagie T, Watemberg N (2002). A normal second-trimester ultrasound does not exclude intracranial structural pathology. Ultrasound Obstet Gynecol.

[38] Eurenius K, Axelsson O, Cnattingius S, Eriksson L, Norsted T (1999). Second trimester ultrasound screening performed by midwives; sensitivity for detection of fetal anomalies. Acta Obstet Gynecol Scand.

[39] Chitty LS (1995). Ultrasound screening for fetal abnormalities. Prenat Diagn.

[40] Viellas EF, Domingues RM, Dias MA (2014). Assistência pré-natal no Brasil [Prenatal care in Brazil]. Cad Saúde Pública.

